# Reoperation as a Result of Raised Intracranial Pressure Associated with Cyst Formation in Tumor Cavity after Intracranial Tumor Resection: A Report of Two Cases

**DOI:** 10.1155/2010/634839

**Published:** 2010-09-28

**Authors:** Jinlu Yu, Wenji Xiong, Limei Qu, Haiyan Huang

**Affiliations:** ^1^Department of Neurosurgery, First Hospital of Jilin University, Changchun 130021, China; ^2^Department of Radiology, First Hospital of Jilin University, Changchun 130021, China; ^3^Department of Pathology, First Hospital of Jilin University, Changchun 130021, China

## Abstract

Reoperation as a result of increased intracranial pressure (ICP) associated with cyst formation in an intracranial tumor resection cavity is a rare clinical condition. We report two cases of reoperation as a result of raised ICP associated with cyst formation in the tumor resection cavity, one arising after glioma resection and the other after meningioma resection. In both cases, a “valve”-like structure was noted intraoperatively in the roof region of the tumor resection cavity. Surgical resection of the “valve”-like structure led to slow regression over several months after the reoperation rather than to immediate disappearance of the cyst. Both cases illustrate that the “valve”-like structure formed in the roof region of the tumor resection cavity may be responsible for cyst formation. Surgical resection of it provides good long-term outcomes in such patients though short-term outcomes are unsatisfactory; we speculate that if the resection of the cortical tissue around the “valve”-like structure is enough wide, its return may be avoided.

## 1. Introduction

Raised intracranial pressure (ICP) is common in patients undergoing intracranial tumor resection. Reoperation as a result of raised ICP is an important prognostic factor, often resulting in a poor outcome. Raised ICP associated with intracranial tumor resection frequently arises as a result of hemorrhage in the tumor resection cavity, peritumoral cerebral contusion, cerebral edema, and/or hemorrhage at adjacent or remote sites [1–3]. In contrast, reoperation as a result of raised ICP secondary to cyst formation in an intracranial tumor resection cavity is a rare clinical condition. In a retrospective analysis of 4,782 patients with intracranial tumor undergoing surgical treatment at our hospital over the past 10 years, we identified 98 patients who underwent reoperation owing to high ICP, and two of them developed high ICP as a result of cyst formation in the tumor resection cavity. Herein, we report the two cases, one arising as a result of repeated cyst formation in the tumor cavity after resection of temporal lobe glioma and the other after resection of sphenoid ridge meningioma. We also discuss the potential mechanisms underlying cyst formation in the intracranial tumor resection cavity.

## 2. Case Presentation

### 2.1. Case 1

**Figure 1:**
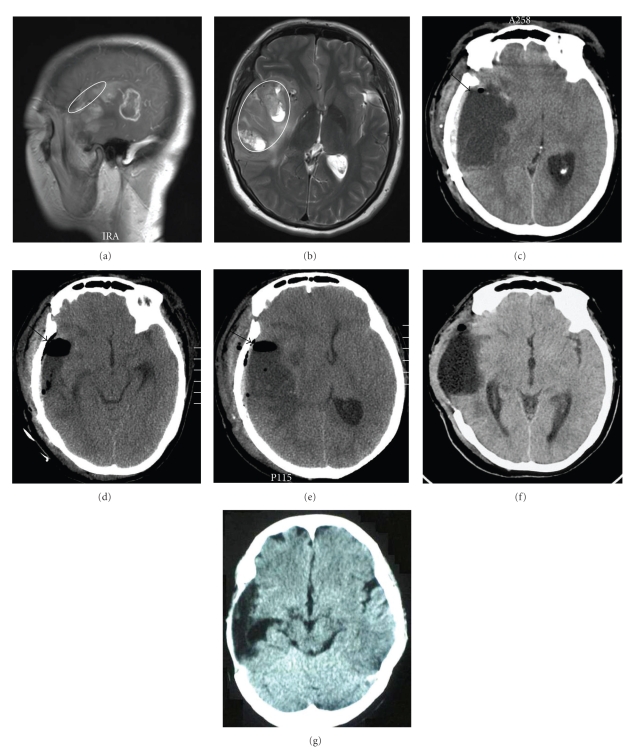
Imaging results for Case 1. (a) Contrast-enhanced MRI showing heterogeneous or ring-like enhancement in the temporal lobe. The elliptical area indicates the cortical incision, via which tumor resection was performed. (b) Axial T2WI showing the tumor resection zone. The cortical tissue located on the surface of the temporal lobe was not resected. (c) CT revealing cyst formation in the tumor resection cavity 3 days after tumor resection. The arrow indicates the position of the “valve”-like structure. (d) Relief of effusion in the tumor resection cavity following the reoperation. The arrow indicates the opening of the roof region of the tumor resection cavity and the disappearance of the “valve”-like structure. (e) Recommencement of effusion in the tumor resection cavity 5 days after the reoperation. The roof region of the tumor resection cavity was sealed again, indicating possible reformation of the “valve”-like structure. (f) Recommencement of effusion in the tumor cavity 1 week after the second reoperation removing the bone flap. (g) Disappearance of cyst formation in the tumor resection cavity 3 months after the second reoperation.

A 44-year-old female patient with a 1-year history of paroxysmal dizziness presented with an exacerbation of dizziness and nausea for 5 days. Physical examination revealed that she had poor memory and computation abilities without other focal neurological signs. A magnetic resonance imaging (MRI) scan of her head revealed a large zone of slightly elongated T1 or shortened T2 signal in her right temporal lobe. After administration of contrast agent, the lesion showed patchy heterogeneous enhancement or ring enhancement (Figures 1(a) and 1(b)). A diagnosis of glioma was considered preoperatively. Surgery was performed via a right frontotemporal approach. Intraoperatively, a pink-purple tumor was noted on the surface of the temporal lobe near the sylvian fissure. The tumor was closely attached to the dura mater, with a soft texture and a rich blood supply. Under an operation microscope, the tumor tissue located adjacent to the sylvian fissure was excised, and a 4 cm cortical incision (as shown in the elliptical area in Figure 1(a)) was made in the sylvian fissure. Further intraoperative exploration showed that the blood supply to the tumor was provided by small branches of the middle cerebral artery. After separating the tumor along the perisylvian region and severing its feeding arteries, the tumor was resected in a piecemeal fashion. The cortical tissue located on the surface of the temporal lobe was not resected (the extent of tumor resection was limited to the elliptical area shown in Figure 1(b)). Surgicel (Ethicon, Inc., a Johnson & Johnson company; Somerville, NJ) was applied to cover the operative field to prevent exudation. The surgery was uneventful, and no openings were found in the cerebral ventricle. Pathological examination demonstrated that the tumor was a grade III oligodendroastrocytoma. The patient recovered well after surgery.

However, 3 days after surgery, she developed somnolence, mydriasis (pupillary diameter of 4.0 mm), disappearance of the light reflex in her right eye, and left limb paralysis. She had a Glasgow Coma Scale (GCS) score of 7. The patient received 250 ml of 20% mannitol and 40 mg of furosemide immediately to lower ICP. A computed tomography (CT) scan of her head revealed a hypodense area in the tumor resection cavity in the right temporal lobe, which led to deformation and dislocation of the surrounding tissue and the cerebral ventricle. Therefore, cyst formation in the tumor resection cavity was indicated (Figure 1(c)). Emergency exploratory surgery was then performed. After incising the dura mater, it was found that the cerebral tissue had an elevated tension. A “valve”-like structure was formed by relocated cerebral tissue, Surgicel, and the arachnoid in the roof region of the tumor cavity, sealing the effusion in the tumor resection cavity (the position of the “valve”-like structure is shown by the arrow in Figure 1(c)). After separating the arachnoid and removing the Surgicel to open the tumor resection cavity, approximately 40 ml of clear, nonyellow liquid was aspirated. As a response to tension reduction, the cerebral tissue collapsed. No communication between the tumor resection cavity and the cerebral ventricle was found. The tumor resection cavity was then washed repeatedly, and the bone flap was returned to its initial location. Postoperative CT reexamination revealed that the space-occupying effect was absent, the roof region of the tumor resection cavity was open, and the “valve”-like structure had disappeared (as shown by the arrow in Figure 1(d)). 

Five days after the reoperation, the patient again developed symptoms of high ICP and cerebral hernia. Repeat CT showed a recommencement of effusion in the tumor resection cavity and resealing of the roof region of the tumor resection cavity, suggesting possible reformation of the “valve”-like structure (Figure 1(e)). A second reoperation was performed to aspirate the effusion at which the sylvian cistern and subarachnoid cistern were fully separated, the “valve”-like structure resected, and the relocated cortex marsupialized so that the tumor resection cavity could communicate with the sylvian cistern and subarachnoid cistern and the effusion could enter the cerebrospinal fluid circulation. Surgicel was not applied this time. Considering that the patient developed cerebral hernia after both operations, the bone flap was removed. The patient recovered well after the second reoperation.

One week later, a CT rescan of her head demonstrated that there was still effusion in the tumor resection cavity, and it was possible that the “valve”-like structure had reformed again (Figure 1(f)). But because the compression on the surrounding tissue was not severe, and considering that the bone flap had been removed and radiotherapy had low risk, the patient was given Gamma Knife therapy immediately after discharge from hospital. Three months later, CT examination showed that the cyst formation in the tumor resection cavity had disappeared, and the tumor had not recurred (Figure 1(g)).

### 2.2. Case 2

**Figure 2:**
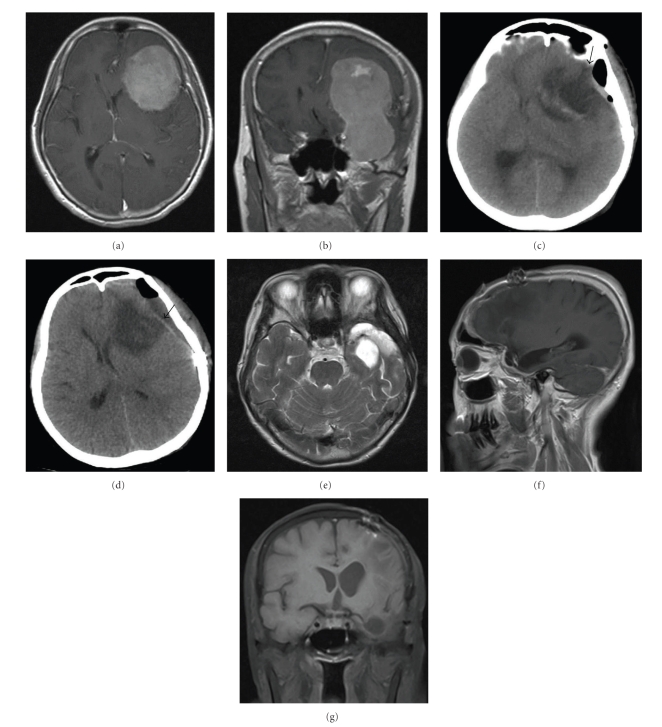
Imaging results for Case 2. (a)-(b) MRI showing sphenoid ridge meningioma. (c) CT revealing cyst formation in the tumor resection cavity 6 days after tumor resection. The arrow indicates the narrow roof region of the tumor resection cavity, where the “valve”-like structure is located. (d) Recommencement of effusion in the tumor cavity 1 week after the reoperation. The arrow indicates that the roof region of the tumor resection cavity became widened. (e)–(g) MRI showing disappearance of cyst formation in the tumor resection cavity 10 months after the reoperation.

A 60-year-old women with a 2-month history of decreased memory presented with an exacerbation and nonfluent speech over the previous month. Physical examination revealed that she had incomplete motor aphasia without other focal neurological signs. An MRI scan of her head revealed a gourd-like area in the left frontotemporal region that spanned the anterior and middle cranial fossa. This abnormal area had a well-demarcated boundary, appearing as a slightly hypointense signal on the T1-weighted image, and as mixed isointense, slightly hyperintense and hypointense signals on the T2-weighted image. The left cerebral ventricle had become narrow as a result of compression. After contrast administration, the lesion showed heterogeneous enhancement (Figures 2(a) and 2(b)). A diagnosis of sphenoid ridge meningioma was considered preoperatively and surgery was performed via a left extended pterional approach. After opening the dura mater, it was found that the grooves of the cerebral cortex near the sylvian fissure had become shallow and showed a pale yellow color. Under an operation microscope, the sylvian fissure was separated, and the inferior frontal gyrus and superior temporal gyrus were retracted to expose the yellowish-white tumor. After partially separating the tumor along its periphery, it was found that the basal part of the tumor was located mainly in the sphenoid ridge and partly at the base of the anterior and middle cranial fossa. Blood supply to the tumor was provided by the small branches of the middle cerebral artery. After severing the feeding arteries, the tumor was totally resected in a piecemeal fashion. The basal dura mater was coagulated following surgery. After the tumor removal, Surgicel was applied to cover the tumor cavity. The surgery was uneventful. Pathological examination demonstrated that the tumor was a meningioma.

Postoperatively, the patient's overall condition was good. However, her condition deteriorated 6 days after the operation. She developed somnolence, mydriasis of her left eye, right limb paralysis, and pathological reflex and had a GCS score of 7, suggesting the formation of a cerebral hernia. To lower ICP, the patient was given 250 ml of 20% mannitol immediately. An emergency CT scan of her head showed a patchy mixed density (mainly hypodensity) area in the tumor resection cavity adjacent to the temporal lobe. The ipsilateral cerebral ventricle was deformed as a result of compression, and the midline structure had shifted toward the right side, suggesting cyst formation in the tumor resection cavity. A band-shaped hyperintense shadow was noted in the intracranial subdural space, in which some shadows of gas density were scattered (Figure 2(c)). Emergency exploratory surgery was performed. After removing a small epidural hematoma and incising the dura mater, it was found that a “valve”-like structure had formed by relocated cerebral tissue, Surgicel, and the arachnoid. The location of the “valve”-like structure was along the sylvian fissure between the inferior frontal gyrus and superior temporal gyrus after the sphenoid ridge (the position of the “valve”-like structure is near the arrow shown in Figure 2(c)) the length along the sylvian fissure was about 5 cm. After separating the arachnoid and removing the Surgicel to open the tumor resection cavity, a virtually clear effusion was noted in the tumor resection cavity in the sylvian cistern. Approximately 30 ml of the effusion was aspirated. As a result, cerebral tissue tension decreased, and the cerebral pulse was good. Subsequent examination revealed that a few blood clots were attached to the base of the tumor resection cavity. After removing the blood clots, no communication was found between the tumor cavity and the cerebral ventricle or cerebral cistern. Subsequently, the sylvian cistern and subarachnoid cistern were fenestrated, and marsupialization of the relocated cerebral tissue was performed so that the tumor resection cavity could communicate with the sylvian cistern and subarachnoid cistern and the effusion could enter the cerebrospinal fluid circulation. The tumor resection cavity was then washed repeatedly, and the bone flap was returned to its initial location. No Surgicel was applied during the second operation.

One week later, a CT rescan of her head revealed return of effusion in the tumor resection cavity, and it was possible that the “valve”-like structure had reformed again. But the roof region of the tumor resection cavity had become wider than before (as shown by the arrow in Figure 2(d)). Since compression on the surrounding cerebral tissue was not severe, a conservative approach was adopted. Ten months later, MRI examination showed that the cyst formation in the tumor resection cavity had disappeared, and the tumor had not recurred (Figures 2(e)–2(g)).

## 3. Discussion

Reoperation after intracranial tumor resection is common in patients with cerebral retraction-induced cerebral edema, cerebral contusion, and/or intracranial hemorrhage, especially in patients with skull base tumors [4, 5]. However, raised ICP or cerebral herniation as a result of the space-occupying effect of cyst formation in a tumor cavity after intracranial tumor resection is rarely encountered in clinical practice. In our retrospective analysis, we found that, among patients undergoing reoperation owing to high intracranial pressure, the rate of reoperation as a result of high intracranial pressure secondary to cyst formation in the tumor resection cavity was approximately 2%. Although intracranial tumor resection cavities filled with fluid are not unusual in patients with large intracranial tumors [6], they often spontaneously diminish or even disappear after cerebral compression is relieved by tumor resection [7]. Cyst formation in an intracranial tumor resection cavity is very rare. 

After intracranial tumor resection, a pale yellow liquid may be produced by the residual tumor or wound surface and accumulate in the tumor resection cavity [8–10]. Such accumulation may occur at an early (average 5.6 weeks) or late (average 3.6 years) stage after tumor resection. Although the space-occupying effect may occur, reoperation owing to development of cerebral hernia is rare. Even if surgery is needed, the prognosis is good, and no repeat cyst formation has been noted [9]. In cases 1 and 2, the liquid aspirated from the tumor resection cavity had a nonyellow color, and cyst formation occurred 3 and 6 days after surgery, respectively, significantly shorter than periods reported in the literature. More importantly, cyst formation recurred rapidly after surgery and regressed over several months. These findings are inconsistent with previous reports mentioned above, suggesting the possible existence of other mechanisms. Cyst formation may also be caused by inflow and accumulation of cerebrospinal fluid in the tumor resection cavity as a result of communication with the cerebral ventricle or cistern [9]. Although the tumors in both case 1 and case 2 were close to the temporal and frontal horns of the lateral ventricle, intraoperative examination and postoperative imaging revealed no communication between the tumor resection cavity and the cerebral ventricle. If communication occurs, the accumulated fluid in the tumor resection cavity may flow back to the cerebral ventricle, which may not give rise to raised ICP. Therefore, the possibility that cyst formation is caused by communication between the tumor resection cavity and the cerebral ventricle can be excluded.

So what mechanism caused the cyst formation? In case 1, an intracerebral operation was performed to remove the temporal lobe glioma. In case 2, resection of the sphenoid ridge meningioma was conducted in the space between cerebral tissue and the dura mater at the skull base; therefore this procedure was an extracerebral operation. Despite large differences in clinical characteristics and surgical procedures, repeated cyst formation in the tumor resection cavity was noted in both cases. Of note, both patients had a large tumor and a large tumor cavity after tumor resection. Exploratory surgery revealed that a “valve”-like structure had formed by relocated cerebral tissue, Surgicel, and the arachnoid in the roof region of the tumor resection cavity. Furthermore, we also found that the liquid aspirated from the tumor cavity had the properties of the cerebrospinal fluid. We therefore surmise that the “valve”-like structure may only permit unidirectional flow of cerebrospinal fluid. When raised ICP occurred, the cerebrospinal fluid entered into the tumor resection cavity via the “valve”-like structure which then sealed the tumor resection cavity, so preventing the effusion from entering the cerebrospinal fluid circulation and resulting in accumulation of liquid in the tumor resection cavity to produce a space-occupying effect. This is similar to the mechanism responsible for the formation of subdural hygroma or iatrogenic cyst [11–14].

**Figure 3:**
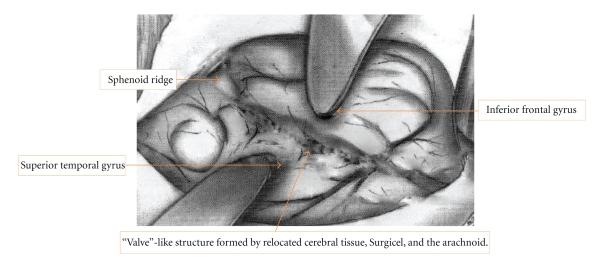
Diagram showing the “valve”-like structure on the surface of the brain.

Rao reported a case of an expanding iatrogenic cyst following temporal lobectomy. In that case, it was proposed that a ball valve obstruction to flow of CSF occurred at the trigone. Ventricular CSF outflow would be forced into the cyst with the increased pressure of respiratory inspiration and cardiac systolic pulses [14]. Kutlay et al. reported a case with iatrogenic arachnoid cyst with distinct clinical picture as a result of bone defect in the floor of the middle cranial fossa, in the case with time CSF entrapment in iatrogenic cyst, which supported the probable existence of a valvular mechanism, leading to the progressive accumulation of fluid within the cyst. Additionally, the cyst itself might have acted as a conductor for transmitting the pulsatile forces of the brain and CSF, and this condition also contributed to enlargement of the cyst [15]. Thus, it is reasonable for us to speculate that the formation of the iatrogenic “valve”-like structure is responsible for cyst formation in the tumor cavity after intracranial tumor resection. The formation of such an unidirectional “valve”-like structure is presumably in part iatrogenic. As the cortical tissue located on the surface of the tumor was not resected, it may provide an anatomical basis for the formation of the “valve”-like structure (see Figure 3).

To prevent return of the effusion in the tumor resection cavity after the reoperation, we aspirated the effusion in the tumor cavity, opened up the communication of the cavity with the surrounding subarachnoid space and cerebral cistern, resected the “valve”-like structure, and marsupialized relocated cerebral tissue so that the effusion could enter the cerebrospinal fluid circulation. However, the immediate postoperative outcome was poor as effusion recommenced in the tumor resection cavity in both cases because of the reformation of the “valve”-like structure. What is the hypothesis about the fact that the cyst postoperatively returns despite the fact that the “valve”-like structure was resected? A retrospective image analysis revealed the presence of edema in the cerebral tissue surrounding the tumor resection cavity. After the “valve”-like structure was surgically resected, edema could push the cortex toward the place of the former “valve”-like structure, which made it reform [16]. 

Therefore, we speculate that the “valve”-like structure may reform as a result of the presence of cerebral edema, and when the roof region of the tumor cavity remains very narrow, the effusion in the tumor resection cavity cannot enter the cerebrospinal fluid circulation, and so it builds up. Subsequent “valve”-like structures were weaker than the earlier ones, so as cerebral edema regresses, the anatomical mechanism underlying the formation of the “valve”-like structure diminishes, allowing the tumor cavity effusion to enter the cerebrospinal fluid circulation efficiently and resolving the effusion. 

Currently there are three main surgical options for the treatment of developmental and iatrogenic cysts: craniotomy with excision of the cyst membrane, CSF drainage (either from the cyst or from the lateral ventricle), and cyst fenestration by means of stereotactic or endoscopic techniques [17–19]. For the cases in this paper, the first option was not feasible because there was no cyst membrane to excise. It was also difficult to excise the cyst since it was not possible to resect the anatomical basis of the “valve”-like structure. Long-term CSF drainage was not possible either due to potential for infection. Thus craniotomy with resection of the “valve”-like structure was the best option, although in this paper the resection of brain tissue around the “valve”-like structure was not enough so that the roof region of the tumor cavity remained very narrow. 

In case 1, postoperative CT reexamination revealed the opening and resealing of the roof region of the tumor resection cavity, suggesting possible disappearance and reformation of the “valve”-like structure (as shown by the arrows in Figure 1). In case 2, postoperative examination showed that the roof region of the tumor resection cavity became widened (as shown by the arrow in Figure 2), and cyst reformation was attenuated, suggesting that the effect produced by the “valve”-like structure is weakened. These imaging changes indirectly confirm our hypothesis that the “valve”-like structure is responsible for cyst formation in the tumor resection cavity. Thus, we speculate that resection of the cortical tissue located on the surface of the tumor to widen the roof region of the tumor resection cavity may avoid the formation of the “valve”-like structure.

## 4. Conclusion

In conclusion, we describe two cases of reoperation as a result of raised ICP owing to cyst formation in the tumor cavity following intracranial tumor resection. We hypothesize that the presence of a “valve”-like structure in the roof region of the tumor resection cavity is responsible for cyst formation in the cavity. Surgical resection of this and marsupialization of the cortex provide good long-term outcomes in such patients though short-term outcomes are unsatisfactory, and we speculate that if the resection of the cortical tissue around the “valve”-like structure is wide enough, the problem may be avoided.
